# Autoreactive T-Lymphocytes in Inflammatory Skin Diseases

**DOI:** 10.3389/fimmu.2019.01198

**Published:** 2019-05-29

**Authors:** Wolf-Henning Boehncke, Nicolo Costantino Brembilla

**Affiliations:** ^1^Department of Pathology and Immunology, Faculty of Medicine, University of Geneva, Geneva, Switzerland; ^2^Divison of Dermatology and Venereology, Geneva University Hospitals, Geneva, Switzerland

**Keywords:** resident memory T-lymphocytes, psoriasis, atopic dermatitis, bullous pemphigoid, pemphigus, alopecia areata, vitiligo, scleroderma

## Abstract

The presence of one or several autoantigen(s) and a response by the adaptive immune system are the key criteria to classify a pathology as an autoimmune disease. The list of entities fulfilling this criterion is currently growing in the light of recent advancements in the pathogenetic understanding of a number of important dermatoses. The role of autoreactive T-lymphocytes differs amongst these pathologies. While they are directly involved as effector cells attacking and sometimes killing their respective target in some diseases (e.g., vitiligo), they provide help to B-lymphocytes, which in turn produce the pathogenic autoreactive antibodies in others (pemphigus and pemphigoid). Atopic dermatits is a chimera in this regard, as there is evidence for both functions. Psoriasis is an example for an entity where autoantigens were finally identified, suggesting that at least a subgroup of patients should be classified as suffering from a true autoimmune rather than autoinflammatory condition. Identification of resident memory T-lymphocytes (T_RM_) helped to understand why certain diseases relapse at the same site after seemingly effective therapy. Therefore, the in-depth characterization of autoreactive T-lyphocytes goes way beyond an academic exercise and opens the door toward improved therapies yielding durable responses. T_RM_ are particularly suitable targets in this regard, and the clinical efficacy of some established and emerging therapeutic strategies such as the inhibition of Janus Kinase 3 or interleukin 15 may rely on their capacity to prevent T_RM_ differentiation and maintenance. Research in this field brings us closer to the ultimate goal in the management of autoimmunity at large, namely resetting the immune system in order to restore the state of tolerance.

## Introduction

**Figure 1:**
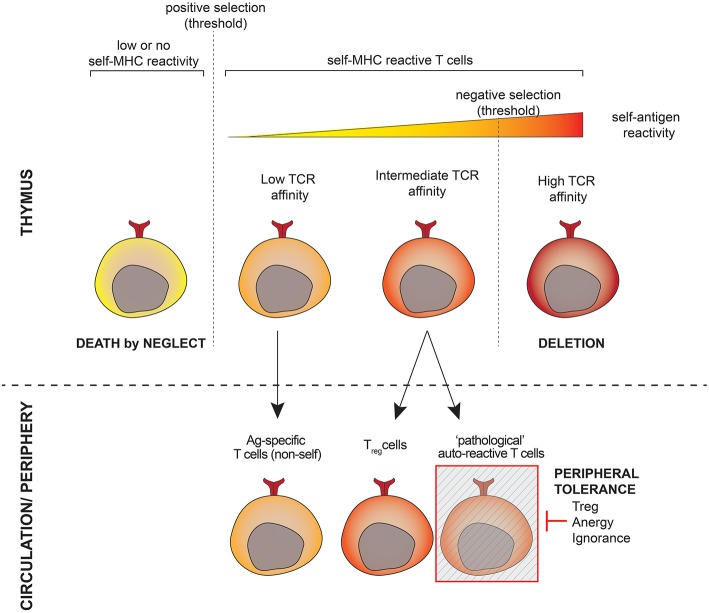
Shaping the T-cell repertoire. T-lymphocytes undergo positive selection to ensure restriction to the host's antigen presenting molecules as well as negative selection to eliminate strongly autoreactive cells in the thymus prior to populating the periphery. T-cells whose TCR affinity is close to the threshold for negative selection have a high potential for autoreactivity and escape thymic negative selection. A subset of these cells is programmed to become T_reg_ cells, others (the potentially pathogeneic autoreactive cells) are kept under the control of the peripheral tolerance.

During T-cell development, T-cell receptors (TCR) are generated randomly and subsequently undergo a selection process in the thymus (Figure 1). This includes a positive round of selection to guarantee selfMHC-restriction and a negative round to eliminate T-cells that recognize self-antigens too strongly. The fate of a developing T-cell depends on the strength and duration of the interaction between its TCR on the one hand and the self-peptide-MHC complexes presented by thymic cells on the other hand. While TCRs with theoretically infinitive affinity may be generated, developing T-cells have to match a binary destiny: life or death. The intrinsic mechanism of selection is thus prone to the development of auto-reactive cells, especially those harboring TCRs with affinity close to the threshold for negative selection (1). Another issue comprises the presence of tissue-specific genes that are not expressed in the thymus and poorly regulated by the autoimmune regulator [AIRE, (2)], toward which T-cells are not depleted during thymic selection. As a result of these mechanisms, autoreactive T-cells are released into the circulation and inevitably exist in all individuals (3). Autoreactive T-lymphocytes behave just like “normal” ones, namely, they recognize antigenic peptides presented to them in the context of a host's antigen presenting HLA molecule and become activated if the appropriate signals are provided. The difference lies in the antigenic peptide, which for “normal” T-lymphocytes is a foreign structure, e.g., part of a pathogen, while autoreactive T-lymphocytes are specific for peptides representing “self,” e.g., part of an anchoring protein of keratinocytes.

The autoreactive compartment comprises at least two types of cells: self-reactive cells programmed during development to control the immune response as a part of a peripheral tolerance mechanism (this is the case for regulatory T-cells, T_regs_), and autoreactive cells that may turn to be harmful and cause autoimmunity. The latter are, however, found at low frequencies in the peripheral T-cell pool, and their functions are hidden by peripheral tolerance mechanisms. Harmful autoreactive T-cells that escape central tolerance are indeed rendered inoffensive via anergy, ignorance, and active suppression by T_regs_ [reviewed in (4)].

T_regs_ can be classified into thymic T_regs_, peripheral T_regs_, and *in vitro* induced T_regs_ (5). These subsets show functional and phenotypic similarities, but differ epigenetically. T_regs_ interact directly with different cell types of the innate and adaptive immune systems, but also exhibit their anti-inflamamtory effects via cytokines such as IL-10, IL-35, TGF-β, and galectin-1.

The frequency of autoreactive T-cells specific for a given self-antigen has been evaluated by peptide-MHC tetramer technology to be similar to those specific for foreign antigens, in the order of 1 to 10 per million T-cells (6). The study of the total autoreactive T-cell repertoire in healthy individuals is, however, hampered by the fact that peripheral tolerance mechanisms make autoreactive T-cells functionally indistinguishable. Richards and colleagues addressed this issue by analyzing the “exposed” self-reactive T-cells upon removal of T_reg_ cells in Foxp3^DTR^ mice. Self-reactivity was observed in about 4% of peripheral CD4+ and CD8+ T-cells, a frequency similar to the responses to allo-MHC complexes or superantigens (7).

Thus, autoreactive T-cells are readily detectable in healthy individuals, but they are efficiently controlled by peripheral tolerance. When the tolerance is broken, autoreactive T-cells may become activated and generate overt autoimmunity. In that regard, interesting insights are being generated by the therapeutical use of checkpoint inhibitors, e.g., CTLA-4 and programmed cell death protein 1 (PD-1) blocking antibodies. These compounds represent a promising approach to treat various cancers since they boost specific anti-tumor T-cell immunity by restraining tolerogenic mechanisms exploited by the tumor. The drawback is that peripheral tolerance is weakened and patients may develop so called immune-related adverse events (irAEs). These irAEs differ from “classic” organ-specific autoimmune disease in as much as they affect a broader range of organs and cells (8). These data clearly show how autoreactive T-cells may be reactivated in particular situations.

How tolerance is broken or evaded during “classical” autoimmunity is a complex and incompletely understood matter. Autoimmune responses are currently thought to arise from a combination of genetic and environmental factors. For example, HLA polymorphisms could result in altered regulation or reduced threshold for autoreactive T-cells, with environmental factors constituting the initial triggering for inappropriate activation (9). Regarding peripheral tolerance, T_regs_ may become dysfunctional through at least 4 distinct mechanisms, namely plasticity (capacity to produce IL-17 after loss of the transcription factor FOXP3), reduced CD18 expression, epigenetic changes, and inhibitory mRNA targeting FOXP3. This allows proinflammatory cells such as Th1 and TH17 lymphocytes to escape regulation and to perform their effector functions in an uncontrolled manner (10).

Activation of autoreactive T-lymphocytes is a key event in almost any kind of autoimmune response: while T-cells are important effectors in some entities (e.g., psoriasis), their principal mode of action in other diseases is to provide help for B-lymphocytes produce the disease-mediating auto-antibodies (e.g., bullous pemphigoid). A clinical consequence is that drugs targeting T-cell function are highly effective to treat the former, while B-cell directed drugs currently represent the gold standard for the latter.

We will now discuss the current pathophysiological concepts of the clinically most relevant inflammatory skin diseases for which a role of autoreactive T-lymphocytes is either well-established or suggested based on the evidence.

## Vitiligo

Vitiligo (11) (Table 1) occurs in about 1% of the population worldwide and is thus the most common cause of acquired skin, hair, and oral depigmentation. It is characterized by the occurence of well-demarked whitish macules. The histology of lesional skin is characterized by a dermal inflammatory infiltrate along with hypopigmentation in the basal layer of the epidermis, the latter being caused by destruction of melanocytes. The hypothesis of it representing a T-cell mediated autoimmune disease is backed up by its HLA association, the identification of melanocyte-derived potential autoantigens (12, 13), and demonstration of CD8+ T-lymphocytes targeting and destroying melanocytes (14). Consequently, most of the strategies used to treat vitiligo have an impact on T-cell function. This holds true for topical glucocorticosteroids and calcineurin inhibitors as well as phototherapies, while the use of other systemic immunosuppressants is not recommended based on current guidelines (15).

**Table 1:** The role of autoreactive T-lymphocytes in inflammatory skin diseases.

**Entity**	**HLA association**	**autoantigen**	**T-cell involvement**
Vitiligo	Yes	Melanocyte-dervied antigens	• CD8+, destroying melanocytes • T_RM_
Alopecia areata	Yes	• Trichohyalin? • Tyrosinase-related protein-2	• CD8 •(CD4+ needed in animal models for maximal induction)
Psoriasis	Yes	• ADAMTS-like protein 5 • (LL-37) • (neolipid antigens)	• CD8+ • T_RM_
Scleroderma	Yes	Unknown (Nuclear antigens (Topo1, RNApolIII, centromere) recognized by autoantibodies)	• CD4+, CD8+
Bullous pemphigoid	Yes	NC16A domain of BP180	CD4+
Pemphigus vulgaris	Yes	Desmoglein 3	CD4+
Atopic dermatitis	No	Hom s 2 (α-NAC)	CD8+, producing IL-4 and IFN-γ

Frequently, rapid recurrences at identical locations are observed after stopping therapy (16), arguing in favor of a persisting cutaneous autoimmune memory, which reactivates the disease once the treatment has been stopped. Resident memory T-lymphocytes (T_RM_) are candidates representing this memory (17), and several groups have described their presence in vitiligo (18–20). Efforts to provide evidence in favor of their direct pathogenetic involvement are hampered, as there are currently no tools at disposition that allow for their specific removal or blockade without affecting other T-cell populations. Recently, Richmond et al. confirmed the T_RM_ phenotype of autoreactive T-lymphocytes within lesions of vitiligo patients (21). Using a mouse model, they went on to demonstrate that blocking the receptor for interleukin-15 (IL-15)—a crucial cytokine for T_RM_ generation and function—with a specific antibody reverses the disease and depletes T_RM_ after long-term therapy. Interestingly, even short-term local intradermal treatment provided durable repigmentation without depletion of autoreactive T-lymphocytes. Taken together, the authors suggest that autoreactive T-lymphcytes are recruited to the skin, encounter IL-15 presented to them by keratinocytes, up-regulate interferon gamma (IFN-γ), and depend on IL-15 for survival once they become resident in the epidermis.

## Alopecia Areata

Alopecia areata (AA) (22) (Table 1) has a cumulative lifetime incidence of about 2%. It manifests in the form of non-scarring, patchy hair loss, with a narrowing of the hair shaft near the scalp as a hallmark. Histology reveals a peribulbar lymphocytic infiltrate, comprising CD8+ T-lymphocytes within the follicular epithelium, and CD4+ T-lymphocytes around the hair follicules. Evidence in favor of an autoimmune pathogenesis comes from its HLA association (23), association with other autoimmune diseases such as vitiligo (24), and response to immunosupressive therapies (25).

Similar to the central nervous system or the placenta, the hair follicule is considered an immune privileged site. Evidence in favor of this hypothesis comes from experiments showing survival of melanocytes in hair follicules grafted from black guinea pig skin onto albino guinea pigs, and viability of human dermal sheath tissues within sex-mismatched transplants (26, 27). It is the loss of this immune privilege that is considered a cornerstone in the pathogenesis of AA. The mechanisms of immune privilege preservation in hair follicles as well as potential causes of its collapse have recently been reviewed elsewhere (28).

Several lines of evidence, including elegant experiments using human scalp explants grafted onto severely immunocompromised mice, point toward CD8+ T-lymphocytes as important effector cells. However, maximal induction of the AA phenotype requires the help of CD4+ T-lymphocytes (29, 30) Using a well-established C3H/HeJ mouse model (31), characterized by spontaneous development of alopecia and recapitulating many pathologic features of human AA, Xing et al. identified a subset of cytotoxic memory T-lymphocytes (CD8+NKG2D+) as the relevant effector cells for the autoimmune response toward the hair follicle, and postulated that IFN-γ secreted from CD8+ T-lymphocytes erases the immune priviledge in the hair follicle, inducing the production of IL-15 and promoting further cellular autoimmunity (32). This hypothesis is strikingly similar to the current pathogenic concept in vitiligo (see above).

Progress toward identifying putative autoantigens in AA was made using a two-step screening approach: first, candidate peptides derived from proteins expressed by keratinocytes or melanocytes (as the suspected targets of autoreactive T-lymphjocytes) were designed *in silico* based on their affinity to the AA-associated allele HLA-A^*^0201. These were then screened for their capacity to activate CD8+ T-lymphocytes from AA patients. Using this approach, the hair follicle antigens trichohyalin and tyrosinase-related protein-2 were identified as putative autoantigens, with trichohyalin being capable of inducing mononuclear cells to secrete proapoptotic factors harmful to hair follicle keratinocytes (33).

## Psoriasis

Psoriasis (34) (Table 1) affects around 2% of the population. The characteristic red scaly plaques, which often occur on elbows, knees, and scalp, but can affect any site of the body, already highlight the two major pathogenetic processes active in parallel in psoriasis, namely inflammation (explaining the redness) and epidermal hyperproliferation (hence the scaly plaque). This is histologically reflected by epidermal acanthosis (thickening of viable layers), hyperkeratosis (thickened cornified layer), and parakeratosis (cell nuclei present in the cornified layer). Rete ridges reach deep into the papillary dermis, resulting in profound indentation of both layers. Within the dermis, blood vessels are dilated, contorted, and reach into the tips of the dermal papillae. Finally, there is a mixed epidermo-dermal mononuclear inflammatory infiltrate rich in T-lymphocytes, along with increased numbers of macrophages, mast cells and neutrophils. The epidermal accumulation of the latter results in the so-called pustules of Kogoj and subcorneal microabscesses (Munro's microabscesses).

Ever since the introduction of ciclosporine A into the therapeutic armentarium, psoriasis has been regarded as a T-cell driven disease, and the important role of T-lymphocytes triggering psoriasis has been shown using T-cell transfer experiments in a xenogeneic transplantation model (35). The observation by Boyman et al. that skin grafts from non-lesional skin of psoriasis patients transferred onto immunodeficient mice spontaneously developed the phenotype of lesional skin was taken as evidence for the presence of T_RM_ (36). A clinical study evaluating E-selectin blockade as a means to prevent the extravazation of T-lymphocytes into the skin showed little therapeutic efficacy in treating psoriasis (37). This was taken as evidence that psoriasis depends primarily on skin resident T-lymphocytes rather than recirculating T-lymphocytes. On the other hand, inhibiting CD8+ T-cell migration into the epidermis blocked the psoriatic transformation of the grafts in the model used by Boyman et al. (38), and the blockade of LFA-1, another adhesion molecule involved in T-cell trafficking into the skin, does show clinical efficacy (39). The discrepancy between the two clinical studies cited here may be explained by the complexity of the interplay of adhesion molecules as well as mediators in the recruitment of lymphocytes into the skin (40). Whether or not interference with lymphocyte recruitment to the skin has a therapeutic efficacy, it may thus depend on the exact molecular target(s) as well as the degree to which functional inhibition of a given adhesion molecule is achieved *in vivo*. The above-mentioned observations support the idea of ongoing psoriasis exhausting the pool of skin-resident pathogenic T-lymphocytes and requiring substitution from lymphoid organs, where these clones might reside as central memory T-lymphocytes (41).

HLA association, namely with HLA class I (42, 43), and the oligoclonality of the T-cell infiltrate (44) have been interpreted as evidence for an antigen-driven, possibly autoimmune pathogenesis. To date, HLA-C^*^06:02 is considered the most important psoriasis risk allele (45). In a series of elegant experiments, Arakawa et al. set out to identify potential autoantigens (46). First, they identified HLA-C^*^06:02 expressing melanocytes as targets for T-cell hybridomas expressing a receptor, which was previously identified as being functionally relevant in patients. They went on to identify the melanocyte ADAMTS-like protein 5 as putative autoantigen and observed T-lymphocytes attacking melanocytes in psoriasis lesions of patients.

Another potential autoantigen LL-37 is generated by extracellular cleavage of a 170 amino acid cathelicidin antimicrobial peptide (47). However, to the knowledge of the authors, it has yet to be shown that the antigen processing and presentation machinery of the target cell is capable of generating an HLA class-I restriced peptide from the original protein. Without such confirmation, a role as autoantigen for CD8+ T-lymphocytes needs to be interpreted with care (48).

Finally, neolipid antigens generated by mast cell phospholipase have been described as targets for psoriatic CD1a restricted T-cells (49).

Besides autoreactive T-lymphocytes, T_regs_ are also thought to contribute to psoriasis through ineffective control of proinflammatory cells: while CD4^+^CD25^+^FOXP3^+^ cells are readily detectable in the blood of psoriasis patients, they are unable to suppress Th1 effects. In contrast, T_regs_ isolated from healthy individuals and co-cultured with Th1 cells from psoriasis patients were able to suppress biological effects of the latter (50). In line with this finding, a positive correlation between T_regs_ and Th17 cells (key effector cells in the context of the psoriatic inflammation) in psoriasis has been shown (51, 52) suggesting that the immune system attempts to downregulate the ongoing inflammation through increased presence of T_regs_, but these are unable to effectively inhibit the disease. As outlined in the introduction, there are at least four mechanisms of rendering T_regs_ dysfunctional.

## Scleroderma

Scleroderma (Systemic Sclerosis, SSc) (53) (Table 1) occurs in around one in 10,000 people worldwide and has a remarkable female predominance. Although rare, scleroderma is associated with high mortality, greater than any other rheumatic disease. Clinical manifestations are heterogeneous, and progression may vary from somewhat stable situations to sometimes rapidly progressive disease. Cases of scleroderma are generally classified into one of two major subsets according to the extent of skin fibrosis: the limited form is defined by skin involvement distal from elbows and knees, whereas patients affected by the diffuse form present extensive cutaneous and internal organ involvement.

The pathophysiology of scleroderma includes three main peculiarities: progressive fibrosis, diffuse fibroproliferative microangiopathy, and inflammation. While the exact etiology remains unknown as of now, environmental as well as genetic factors are considered to be important in the initial phase of the disease. Several lines of evidence suggest that autoreactive T-cells and chronic inflammatory events participate in the initiation or maintenance of the fibrotic process (54).

The first hint comes from genetic and epigenetic studies. Loci in the HLA class II region show the strongest genetic association with the disease, followed by genes involved in B- and T-cell activation and innate immunity. Similarly, epigenetic alterations generally include genes with a role in autoimmunity and T-cell function or regulation (55).

Next, assessment of scleroderma skin revealed that a mononuclear cell infiltrate precedes the fibrotic change and that synthesis of collagen is maximal above it (56, 57). Interestingly, T-cells infiltrating the affected skin show a limited TCR usage, suggesting that they have undergone clonal expansion in response to a specific autoantigen (58).

Another aspect underlying the autoimmune nature of scleroderma is the characteristic presence of anti-nuclear antibodies, including anti-centromere, anti-topoisomerase 1 (anti-topo1) and anti-RNA polymerase III (RNApolIII) antibodies. Anti-topo1 antibodies have high specificity for scleroderma (20–40% of patients) and predict more severe disease and mortality (59). Confirming the involvement of autoreactive T cell-dependent B-cell help, anti-topo1 antibodies are known to exhibit class switching and show strong associations with specific HLA alleles (60). Seminal works from Boin and Wright's groups proved that tolerance to topo1 antigens is effectively broken and topo1-autoreactive T-cells are found in patients presenting these antibodies (61, 62). The possible pathogenic role of nucelar antibodies remains, however, debated, while the frequency of circulating autoreactive CD4+ T-cells was shown to predict interstitial-lung disease development (62).

Regarding the presence of autoantibodies, a striking feature is that patients having anti-RNApolIII antibodies but not the other types show a temporal clustering between the onset of cancer and scleroderma. Rosen et al. provided some evidence in favor of a causative link and proposed that somatic mutation of the RNApolIII in the tumor may actually trigger the activation of autoreactive T-cells that cross-react with the tumor mutated antigen, leading to the initiation of the autoimmune scleroderma response (63).

Finally, three randomized controlled trials tested the efficacy of autologous stem cell transplantation for treating scleroderma. A durable improvement in skin fibrosis, pulmonary functions and quality-of-life measures were achieved, suggesting that immune “replacement” and thus deletion of possible autoreactive lymphocytes might be beneficial (64).

While autoreactive T-cells likely represent an integral or causative part of the pathogenesis of scleroderma, the autoantigens and the mechanisms involved remain largely unknown.

Besides autoreactive T-cells, the quantification of T_regs_ in patient tissues generated contradictory results: most studies reported a reduced frequency while some found an increase in T_reg_ numbers, particularly in early and active disease. Nonetheless, T_regs_ appear to consistenly harbor a defective suppressing capacity in scleroderma, leading to a weaker peripheral tolerance (65, 66).

## Bullous Pemphigoid

Bullous pemphigoid (BP) (67) (Table 1) develops in 12 to 66 individuals per million people per year. It is characterized by tense blisters and erosions, often preceded by urticarial lesions. Blisters often arise on the flexor sites of the extremities as well as the trunk; they may persist for several days before transforming into secondary lesions, namely erosions and crusts. Histopathology of lesional skin specimen shows subepidermal splitting, leaving the dermo-epidermal junction intact, and a dense infiltrate rich in granulocytes, primarily eosinophils and neutrophils.

Contrary to the entities discussed so far, BP is considered an antibody-mediated autoimmune disease. It is characterized by immunoglobulin (Ig) G and E autoantibodies recognizing BP230 and BP180, both antigens localizing to the hemidesmosomes. While BP230 represents an intracellular component, BP180 is a transmembrane glycoprotein of keratinocytes constituting the basal layer. The collagen type XVII extracellular NC16A domain of BP180 comprises immunodominant B-cell epitopes. Numerous *in-vitro* experiments as well as animal models have established a pivotal role for Fc receptor-mediated effects in the process of blister formation (68).

Repetitively, reports on a possible involvement of CD4+ helper T-lymphocytes were published (69, 70). However, while the key role of autoantibody responses to BP180 is well-established in BP, the evidence and clinical relevance of disease-specific T-lymphocyte responses remained unclear. More recent work by Pickford et al. shed more light on the T-lymphocyte participation in BP autoreactivity (71). They tested proliferative and cytokine responses of peripheral blood mononuclear cells from patients and controls to recombinant NC16A and a panel of overlapping peptides spanning this region of BP180. They identified numerous disease-associated factors which influence the composition of the cytokine responses. The strongest association with BP was observed for specific IL-4 and IgE responses, suggesting a potential role for autoreactive Th2 lymphocytes. These results align well with older observations using a humanized mouse model, where the importance of NC16A specific Th-lymphocytes was demonstrated (72, 73). Interestingly, this NC16A response by T-lymphocytes was restricted to HLA-DQB1^*^0301, a known BP susceptibility allele (74).

A role of T_regs_ in BP was recently documented by Haeberle et al. in a mouse model for BP: they showed that autoantibodies against different known autoantigens, including BP230, develop spontaneously in T_reg_ deficient scurfy mice, leading to blister formation. This study suggests that autoreactive T-lymphocytes initiate the disease in the absence of proper immune control (75).

## Pemphigus Vulgaris

Pemphigus vulgaris (PV) (76) is another blistering autoimmune disease with an immunopathogenesis comparable to pemphigoid. Its incidence is in the order of 1–5 new cases per million people per year. Clinically, PV often starts progressively with mucosal lesions and subsequently extensive flacid skin blisters. Blister formation occurs intraepidermally. Autoreactive IgG antibodies recognize desmoglein (Dsg) 3 and 1 within desmosomes, adhesion complexes between keratinocytes. HLA-DRB1^*^04:02 and HLA-DQB1^*^05:03 have been identified as disease-associated HLA class II alleles.

Similar to BP, a role for autoreactive helper T-lymphocytes has long been postulated in PV, but was difficult to demonstrate. Several years ago, Emig et al. showed in a humanized HLA-DRB1^*^04:02 transgenic mouse model that T-lymphocytes recognize human desmoglein 3 epitopes in the context of HLA-DRB1^*^04:02 leading to the induction of pathogenic IgG autoantibodies, which in turn trigger intra-epidermal blister formation (77). Activation of Dsg3-reactive CD4+ T-lymphocytes is restricted to the HLA-DRB1^*^04:02 allele. IgG autoantibodies are produced following CD40-CD40L-dependent T-cell—B-cell interactions and exhibit specificities to both N- and C-terminal epitopes of the human Dsg3 ectodomain. Since then, additional evidence has been generated for an active role of CD4+ helper T-lymphocytes (78), but functional studies in patients are still lacking, and most groups working in the field do not assign a high priority to this aspect of translational research in PV (79).

## Atopic Dermatitis

Atopic dermatitis (AD) (80) (Table 1) has a lifetime prevalence in the order of 10–20% in developed countries with pruritus being the leading symptom. Eczematous lesions can be acute, subacute or chronic, and predilection sites change with age. Co-occurrence of altered epidermal structure and function on the one hand and cutaneous inflammation triggered by pathologic immune responses to antigens encountered in the skin on the other hand are characteristic for AD.

Numerous cell types contribute to antigen presentation in AD, including dermal dendritic cells, epidermal Langerhans cells, and inflamamtory dendritic epidermal cells expressing a high-affinity receptor for IgE (81). The latter explains how these cells can present allergens typically triggering type-I (immediate-type) allergic reactions, subsequently inducing T-lymphocyte mediated type-IV (delayed-type) reactions.

Autoreactivity is a known phenomenon in atopic dermatitis. A systematic review provided evidence that up to a third of patients exhibit autoreactive IgE antibodies (82). This may be based on molecular mimicry, as several IgE-binding keratinocyte-derived antigens show homology with environmental allergens (83). Besides, reports on autoreactive T-lymphocytes date back to the beginning of this decade (84, 85). Based on these observations, which suggested a role for the autoallergen Hom s 2, the α-chain of the nascent polypeptide-associated complex (α-NAC), Roesner et al. performed an in-depth analysis of α-NAC specific CD8+ T-cell responses (86). They found higher numbers of α-NAC specific, terminally differentiated peripheral T-lymphocytes in sensitized atopic patients compared with non-atopic controls. These cells secrete IL-4 and IFN-γ, suggesting a pathogenic role in AD.

As in psoriasis, there is also evidence for substantial dysregulation of T_regs_ in atopic dermatitis. The positive correlation of these cells with disease severity both in mouse models as well as in patients (87, 88) documents the immune system's attempt to downregulate the ongoing inflammation through increased presence of T_regs_, which, however, are unable to inhibit the disease.

## Therapeutic Perspectives

So far, we have reviewed the established or potential role of autoreactive T-lymphocytes in a number of different inflammatory skin diseases. These T-lymphocytes fall principally into two major categories: (1) they function either as effector cells or represent the immunological memory in the skin that may be responsible for relapsing disease or (2) they provide help to B-lymphocytes to produce the pathogenic autoantibodies, as is the case of BP and PV. In atopic dermatitis, there is evidence for both roles.

To date, targeted therapies such as biologics or small molecules allow to directly and specifically interfere with the central pathomechanisms. These therapies are often more effective when compared with conventional anti-inflammatory therapies, making these agents the key compounds of current treatment algorithms (89, 90). However, despite therapeutic effects, which sometimes go way beyond the respective molecules' half-lifes (91), all of these currently available therapies cannot prevent relapse, once they are stopped. Discussions to which extent such currently available therapies are disease-modifying, as we increasingly witness in the context of company-sponsored projects, e.g., in the field of psoriasis (92), seem therefore premature. Instead, remission might be a more appropriate term in this context.

As pointed out in the different chapters above, T_RM_ are a particularly important subpopulation in the pathophysiology of several autoimmune skin diseases. Their presence elegantly explains recurrance of such diseases after seemingly successful therapy. This makes T_RM_ attractive targets to achieve durable responses. Boniface and Senechal recently pointed out potential strategies in this regard, looking at vitiligo as a model for a skin memory disease (93) (Table 2):

**Table 2:** Innovative therapeutic strategies exploring the role of T-lymphocytes.

**Target**	**approach**	**Evidence**	**References**
T_RM_ differentiation and maintenance	JAK3 inhibition	Clinically effective in vitiligo, alopecia areata	(23, 94, 95)
T_RM_ differentiation and maintenance	Anti IL-15 antibodies	Effective in a mouse model of vitiligo	(21)
Preventing T_reg_ transformation	Anti IL-23	Clinically effective in psoriasis	(91)
Autoreactive B-lymphocytes	Chimeric antigen receptor T-lymphocytes	Effective in a mouse model of pemphigus vulgaris	(96)
Immune reconstitution	Multiple • Chemotherapies • Depleting antibodies • AHSCT	Clinically effective in multiple sclerosis, scleroderma	(64, 97, 98)
T_reg_ expansion	mTOR inhibition	Clinically effective in lupus erythematosus, psoriasis	(99–101)

*AHSCT, autologous hematopoietic stem cell transplantation*.

- Strategies to prevent T_RM_ differentiation and maintenance in the skin could target relevant mediators of this process, including IL-15, as discussed above. While effective depletion of skin-resident T_RM_ through direct blockade of the IL-15 receptor has been shown in a mouse model (21), data from patients are not yet available. Another way to inhibit the IL-15 signaling pathway is through inhibition of Janus Kinase 3 (JAK3). Noteworthy, a clinical trial demonstrated the efficacy of the non-specific JAK inhibitor tofacitinib in combination with phototherapy to treat vitiligo (95).
- Next, strategies to prevent accumulation of T_RM_ in the skin might be warranted. As T_RM_ are likely to accumulate after repetitive flares of the disease, maintenance therapies after successful initial disease control could be an adequate approach. In line with this hypothesis, maintenance therapy using a topical calcineurin inhibitor twice weekly in patients with repigmented lesions reduces the recurrance of old vitiligo lesions (16).
- Finally, while T_RM_ depletion remains to be demonstrated in patients, it seems feasible to aim at dampening T_RM_ activation through the long-term use of immunomodulating agents. In fact, both clinical studies cited above provide evidence for the efficacy of such an approach (16, 95). The study by Liu et al. assessed the effects of JAK inhibition and phototherapy separately, allowing for the conclusion that phototherapy seems necessary for melanocyte regeneration, while JAK inhibition suppresses T-cell mediators of vitiligo (95).

Noteworthy, the current pathogenetic concept of AA also suggests a role for memory T-lymphocytes, IFN-γ, and IL-15 (32). In that very publication, the authors went on to demonstrate therapeutic efficacy of the non-specific JAK inhibitor tofacitinib as well as the JAK1/2 inhibitor ruxolitinib not only in their mouse model, but also in all three AA patients treated with ruxolitinib (32). As in vitiligo, therapeutic application of JAK inhibitors is currently being explored further in AA (94).

Turning to bullous autoimmune diseases, engineering chimeric antigen receptor (CAR) T-lymphocytes may bear a potential (Table 2). This approach proved effective in leukemia, where a CD19-specific CAR, composed of an extracellular single-chain variable fragment antibody fused to cytoplasmic signaling domains, triggers cytotoxic T-cell reactions upon contact with CD19+ B-lymphocytes, which results in specific and permanent elimination of the respective B-lymphocytes, yielding durable remission (102). Ellebrecht et al. applied this concept to PV, suggesting that expression of Dsg3 as the extracellular domain of a CAR should specifically target cytotoxic responses to those B-lymphocytes bearing anti-Dsg3 B-cell receptors; this should result in a targeted therapy avoiding general immunosuppression (96). Using a mouse model, they succeeded to show that such cells expand, persist, and specifically eliminate Dsg3 autoreactive B-lymphocytes.

While the strategies discussed so far document the substantial efforts to attack pro-inflammatory elements, much less studies assess the potential to strengthen regulatory elements. In this regard, recent observations in the field of psoriasis are of interest. A much cited hypothesis regards the IL-23/IL-17 axis as central in its pathogenesis, with IL-23 triggering the production of IL-17A in numerous cell types, mainly T-lymphocytes; IL-17A is itself a valid target for highly effective therapies (34, 103). As IL-23 inhibition exhibits long-lasting therapeutic efficacy way beyond the half-life of the biologics used (91), one might speculate that additional effects beyond the postulated “upstream” inhibition of IL-17A production might be clinically relevant. T_regs_ are potential targets in this regard. These lymphocytes exhibit substantial plasticity. They can lose their immunosuppressive function under the influence of pro-inflammatory cytokines such as IL-23, IL-1β, and IL-2 through the loss of FOXP3, ultimately switching toward a pro-inflammatory function (104). IL-23 blockade may hamper this transformation. In line with this concept is the observation by Maxwell et al. who used a mouse model of colitis to show that a regulatory, anti-inflammatory environment in the gut can be promoted through blockade of IL-23 (105). Investigating the role of regulatory elements as potential therapeutic targets may deserve more attention in the future (10).

The “holy grail” of treating autoimmune diseases is the re-establishment of peripheral tolerance, may be best reflected by the concept of immune reconstitution therapy (Table 2). Multiple sclerosis has become a model disease in this regard. Numerous approaches have been explored in this disease to “reset” the immune system, including pulsed lympho- or myeloablative treatments through chemotherapies, monoclonal antibodies such as alemtuzumab, and autologous hematopoietic stem cell transplantation (AHSCT). The pivotal trial documenting feasibility of AHSCT in multiple sclerosis was published in 1997 (97). The current status of this field of research has recently been reviewed in this journal (98), supporting the concept of re-establishing a state of peripheral tolerance through deletion of pathogenic clones, achievable via direct ablation in combination with inducing lymphopenia, the latter favoring replicative senescence and clonal attrition. Normalization of genetic signatures along with altered regulatory T-cell populations provide evidence that a restoration of the regulatory network of the immune system really takes place. Moreover, AHSCT is likely to initiate a “rebooting” of the intrathymic selection program that potentially results in the regeneration of a diversified repertoire of naïve T-cells that will again be capable of appropriately modulating immune responses to future antigenic encounters. Similar observations have been made in the field of sceroderma (64).

An alternative approach is emerging in rheumatology, but could also be explored in the inflammatory skin diseases discussed above. This strategy is based on the role of the mechanistic target of rapamycin (mTOR) as a central regulator of T-cell lineage specification (106). Both mTOR complexes 1 (mTORC1) and 2 (mTORC2) need to be blocked simultaneously to allow T_reg_ differentiation. Studying systemic lupus erythematosus (SLE), Kato and Perl showed that IL-21, identified as a key proinflamamtory cytokine in this disease, stimulated both TORC-1 and−2, and abrogated differentiation as well as function of T_regs_ along with autophagy, a phenomenon underlying T_reg_ dysfunction in SLE (99). In turn, dual blockade of TORC-1 and -2 by 4 weeks of rapamycin treatment induced autophagy and corrected T_reg_ function. Progressive improvement in disease activity, associated with a correction of pro-inflamamtory T-cell lineage specification was also observed in in patients with active SLE during 12 months of sirolimus treatment (100). This approach might have the potential to restore tolerance in other autoimmune diseases as well, including psoriasis (101).

In summary, it is evident that there are multiple pathways through which autoreactive T-lymphocytes contribute to the pathogenesis of inflammatory skin diseases, some of which have only recently been identified as classical autoimmune disorders. In all of these diseases, targeting the autoreactive T-cell subpopualtion is a promising and in some cases already well-estabilshed therapeutic strategy. T_RM_ are particularly suitable targets to ensure long-lasting therapeutic effects; the same holds true for the strategies that strengthen the immunosuppressive functions of T_regs_. Observations in multiple sclerosis and scleroderma suggest that resetting the immune system in order to restore the state of tolerance as the ultimate goal in the management of autoimmunity at large may be feasible.

## Author Contributions

W-HB and NB jointly selected the topic for the review, performed the literature review, wrote the manuscript, and designed the figure along with tables.

### Conflict of Interest Statement

The authors declare that the research was conducted in the absence of any commercial or financial relationships that could be construed as a potential conflict of interest.
